# Social Media Use and Oral Health–Related Misconceptions in Saudi Arabia: Cross-Sectional Study

**DOI:** 10.2196/70071

**Published:** 2025-02-10

**Authors:** Rahaf Hamdan BinHamdan, Salwa Abdulrahman Alsadhan, Arwa Zohair Gazzaz, AlBandary Hassan AlJameel

**Affiliations:** 1 Department of Periodontics and Community Dentistry College of Dentistry King Saud University Riyadh Saudi Arabia

**Keywords:** social media, oral health, health misinformation, digital health, Saudi Arabia, public health, Instagram, Snapchat, TikTok, Twitter

## Abstract

**Background:**

Social media has become a central tool in health communication, offering both opportunities and challenges. In Saudi Arabia, where platforms like WhatsApp, Snapchat, and Instagram are widely used, the quality and credibility of oral health information shared digitally remain critical issues. Misconceptions about oral health can negatively influence individuals’ behaviors and oral health outcomes.

**Objective:**

This study aimed to describe the patterns of social media use and estimate the prevalence of oral health–related misconceptions among adults in Saudi Arabia. Additionally, it assessed the associations between engagement with oral health information, self-reported oral health, and the presence and count of these misconceptions.

**Methods:**

A cross-sectional survey was conducted over 10 weeks, targeting adults aged 15 years and older in Saudi Arabia. Data were collected from a total sample size (n=387) via a questionnaire distributed through targeted advertisements on Instagram, TikTok, Snapchat, and X (Twitter). The prevalence of oral health–related misconceptions was estimated using descriptive statistics, including counts and percentages. Chi-square tests described sociodemographic, social media engagement, and self-reported oral health. Logistic and Poisson regression analyses were used to assess associations between engagement and self-reported oral health with misconceptions. Logistic regression models provided odds ratios and adjusted odds ratios with 95% CI to assess the presence of oral health misconceptions. Poisson regression was used to calculate mean ratios and adjusted mean ratios (AMRs) for the count of misconceptions.

**Results:**

WhatsApp (n=344, 89.8%) and Instagram (n=304, 78.9%) were the most frequently used social media platforms daily. Common oral health misconceptions included beliefs that “Pregnancy causes calcium loss in teeth” (n=337, 87%) and “Dental treatment should be avoided during pregnancy” (n=245, 63.3%). Following dental-specific accounts was significantly associated with lower odds of having any misconceptions (adjusted odds ratio 0.41, 95% CI 0.22-0.78) and a lower count of misconceptions (AMR 0.87, 95% CI 0.77-0.98). Conversely, trust in social media as a source of oral health information was associated with a higher count of misconceptions (AMR 1.16, 95% CI 1.02-1.31).

**Conclusions:**

Social media platforms are essential yet double-edged tools for oral health information dissemination in Saudi Arabia. Participants who followed dental-specific accounts had significantly lower misconceptions, while trust in social media as a source of information was linked to higher counts of misconceptions. These findings highlight the importance of promoting credible content from verified sources to combat misconceptions. Strategic collaborations with dental professionals are necessary to enhance the dissemination of accurate oral health information and public awareness and reduce the prevalence of oral health–related misconceptions.

## Introduction

We live in a new era filled with rapid technological advancements that facilitate everyday life. The internet has been used worldwide for many purposes, and it has become an important tool that can improve the distribution of information and health care delivery and outcomes [1]. Web 2.0 is the second generation of the World Wide Web, emphasizing the shift from static web pages to more dynamic and participatory content, with social media becoming a key element shaping daily life [2]. Globally, social media platforms such as Facebook, Twitter, YouTube, and WhatsApp allow individuals to access and share information at a unique scale [3-5]. In Saudi Arabia, platforms like WhatsApp, Snapchat, and YouTube are heavily used for general communication and increasingly for seeking health-related information [6].

Social media platforms have emerged as powerful tools in health communication, offering both opportunities and challenges. In oral health, social media platforms can provide greater access to oral health information, engaging educational content, and web-based interaction with dental professionals [7,8]. For example, YouTube has been identified as a valuable resource for educating patients with leukemia on oral care [9]. Similarly, Snapchat-based interventions have effectively enhanced oral health knowledge among pregnant women in Saudi Arabia [10]. Oral health promotion campaigns delivered through social media platforms have been shown to improve oral health outcomes [11,12].

Despite social media benefits, information quality and reliability with sources often lack clear authorship or transparency, leading to difficulty in assessing information credibility for users [13-16]. This is particularly problematic in oral health, where misinformation—false or inaccurate health claims—could lead to negative oral health outcomes if it was taken by the users and distributed without consulting an expert [17,18]. Disinformation—information deliberately spread with the intent to harm—exacerbates these risks [19]. For example, practices such as using oil pulling to reduce mouth bacteria and prevent dental caries or relying on home remedy pastes to alleviate tooth pain are often shared digitally without scientific evidence [20]. This study uses “misconception” as a comprehensive term to encompass these various forms of inaccurate or misleading oral health information.

In addition to content concerns, sex-based differences in health behaviors, perceptions, and information-seeking patterns play a role in how individuals engage with oral health information on social media. It has been shown that women are more likely to seek web-based health information and engage with health care services compared to men [21]. They are also more likely to engage with and trust content shared on social media, making them a key demographic for addressing oral health misconceptions through these platforms [22]. Conversely, men may be less likely to search for web-based health information or disease prevention, which may lead to the persistence of misconceptions about oral health practices [23]. Sex differences explain that while women may be more susceptible to health misinformation due to their higher engagement, men may require distinct strategies to capture their attention and promote oral health awareness [24]. Such differences suggest that sex may influence the way individuals access and interpret oral health–related information on social media.

In Saudi Arabia, where 99% of the population are internet users actively engaging with social media platforms, this widespread use facilitates the diffusion of oral health misconceptions, leading users to accept inaccurate information and challenge health care professionals [25]. The challenge lies in the sheer quantity and variable quality of information available on social media platforms, which could lead to negative oral health outcomes if one misconception keeps spreading. For example, widespread beliefs about ineffective home remedies and misconceptions about neglecting primary teeth, dental treatment, and oral care during pregnancy continue to be prevalent among people in Saudi Arabia [26-28]. These misconceptions lead to public health implications, as untreated oral health issues can exacerbate systemic health conditions and increase health care costs [29]. To our knowledge, no previous study has specifically explored the context of oral health misconceptions on social media within this population, highlighting the need for this study.

This study aims to describe the patterns of social media use and to estimate the prevalence of oral health–related misconceptions among adults in Saudi Arabia. Additionally, the study aims to explore how social media engagement influences oral health misconceptions, with results stratified by sex to explore potential differences.

## Methods

### Study Design

This study is a cross-sectional survey. We collected the data over a 10-week period from September 9, 2024, to November 17, 2024, using an Arabic-language questionnaire developed on SurveyMonkey (SurveyMonkey Inc) among adults aged 15 years and older residing in Saudi Arabia. The questionnaire was initially developed in English and translated into Arabic to suit the target population. The questionnaire link was distributed through targeted web-based advertisements on the most popular social media platforms in Saudi Arabia [6], including Instagram, X (Twitter), Snapchat, and TikTok. Participants clicking on the advertisements were redirected to a dedicated web page containing a detailed study description and consent form, followed by the questionnaire. At the start of the survey, an introductory statement provided information on the study objectives, assured confidentiality, and included the researcher’s contact information. We obtained the consent via an “Agree” button, which participants clicked to access the survey. Participants were informed that the survey would take approximately five minutes to complete. They were assured that participation is entirely voluntary, with the option to skip questions or withdraw at any time.

### Sample Recruitment

The study was conducted in a digital setting with an open survey accessible to anyone who clicked on the advertisements, using widely used social media platforms in Saudi Arabia (Instagram, X [Twitter], Snapchat, and TikTok) for recruitment. These platforms were chosen due to their high penetration rates and active use in Saudi Arabia [6], ensuring ease of access and effective targeting through their advertising systems. We designed the social media–targeted advertisements to be culturally appropriate and visually engaging, highlighting the voluntary nature of participation and the study’s focus on oral health–related information. The study used a simple random sampling approach facilitated by the advertisement algorithms. These algorithms were used to define the target group—adults aged 15 years and older residing in Saudi Arabia—ensuring that all individuals within this demographic had an equal chance of seeing the advertisement. According to 2022 population data from the General Authority of Statistics (GASTAT) [30] and 2023 internet penetration figures from the Communications, Space, and Technology Commission [6], the target population was estimated at 24,045,450 individuals, representing 99% of the total population in Saudi Arabia who are internet users within the targeted age group. This recruitment strategy aimed to: ensure representation of the population of interest by using an algorithm without prespecified interests, thereby targeting all individuals within the defined demographic; and maximize geographical diversity by specifying the algorithm to reach individuals residing in Saudi Arabia.

However, it is important to acknowledge that participation was voluntary, introducing self-selection bias that may affect the sample’s generalizability. Despite this, the approach was a practical and effective method for recruiting a large, diverse population.

### Effectiveness of the Selected Social Media Platforms

To effectively reach a broad and diverse sample, we launched advertisement campaigns across Instagram, X (Twitter), Snapchat, and TikTok. User interactions were monitored throughout the campaigns to evaluate reach and sample representativeness. Snapchat and TikTok provided us with strategic support to optimize campaign strategies. Snapchat emphasized targeting mechanisms such as predefined and custom audiences, advanced demographics, and accurate algorithmic delivery, which enhanced the ability to reach users based on demographics, location, and language. TikTok highlighted its robust targeting tools, including lookalike or custom audiences, ensuring advertisements were delivered to relevant users while monitoring user interactions like click-through and conversion rates. Although Instagram and X (Twitter) did not provide direct support, their platforms offered advanced targeting capabilities via Ads Manager tools. These tools allowed for audience segmentation by age, location, and sex, enabling effective recruitment across diverse demographics.

### Sample Size

To estimate the prevalence of oral health–related misconceptions, with a 5% margin of error and a 95% CI, a sample size of 384 participants was required (assuming a 50% prevalence to maximize sample size requirements). Anticipating a low participation rate typical of web-based surveys, we assumed a 2% participation rate, leading us to target the reach of approximately 19,200 users in our advertisement campaigns across all platforms to ensure the required sample size. Reach refers to the estimated number of users who would see the advertisement, with metrics from Instagram, X (Twitter), Snapchat, and TikTok helping us track both views and link clicks.

### Selection Criteria

We included both male and female participants aged 15 years and older residing in Saudi Arabia and those who provided informed consent to participate in the study. The age threshold of 15 was selected based on the classification by the GASTAT in Saudi Arabia, which classifies individuals aged 15 years and older as adults for statistical and demographic purposes [31]. Additionally, adolescents in this age group are active users of social media and are likely to be exposed to oral health information digitally, making their inclusion essential to assess the study objectives.

### Questionnaire

We structured the questionnaire into five domains with a total of 29 fixed items distributed across six screens designed to align with the study’s objectives and draw on previously published and validated cross-sectional studies. Face validity was established through expert review by a panel of dental public health specialists. Experts assessed the clarity, relevance, and domain alignment of each item, as well as the overall comprehensiveness of the questionnaire within the study’s objectives. We implemented minor revisions to item wording and categorization based on their feedback (Multimedia Appendix 1). No adaptive questioning was used; all items required mandatory responses prior to proceeding to subsequent sections pages. Respondents could review and modify their answers using the “Back” button.

### The Patterns of Social Media Use

The first domain assessed participants’ use patterns and preferred platforms with one item [6,32-35]. Participants were asked to select their preferred social media platforms across the popular platforms in Saudi Arabia, which included X (Twitter), Snapchat, WhatsApp, Instagram, Facebook, TikTok, Telegram, YouTube, Line, and LinkedIn [6]. Responses were categorized as “more than once a day,” “once a day,” “2-3 times a week,” “4-6 times a week,” “once per week” or “once per month,” and “I don’t use this platform.”

### Engagement With Oral Health Information

The second domain, containing 6 items, focused on the engagement with oral health information obtained from social media [6,32,33,36,37]. Participants were asked binary (yes or no) questions, including whether they “use social media to search for oral health information,” “ever received oral health info from nondental professionals on social media,” “following dental-specific accounts on social media,” “trust in oral health information from social media,” and “influence their trust by social media profile content.” Perception of social media as a good source for oral health information was assessed with response options: “yes,” “sometimes,” and “no.” We dichotomize it to combine “yes” and “sometimes” into a single category representing participants who perceived social media as a good source of oral health information.

### Self-Reported Oral Health

To capture self-reported oral health, we included a single item in the third domain using the Global Self-Ratings of Oral Health question for participants to rate their own oral health status on a 3-point Likert scale as “good,” “average,” and “poor.” [38]. For analysis, self-reported poor oral health was defined as participants selecting “poor,” while better oral health perceptions included those reporting “good” oral health. Participants who neither rated their oral health as good nor poor, reflecting a moderate perception of their oral health, included those reporting “average” status.

### Oral Health–Related Misconceptions

The fourth domain presented 14 statements on common oral health misconceptions in Saudi Arabia, derived from literature [27,28], with response options of “agree,” “disagree,” and “don’t know.” We combined responses of “disagree” and “don’t know” into a single category representing participants who do not believe in the misconceptions. The category “agree” includes those who do agree with these misconceptions.

### Sociodemographic Factors

In the fifth domain, we collected sociodemographic information across seven items: age, which was categorized into five groups: 15-24, 25-34, 35-44, 45-54, and 55+ years; sex; education, which was classified into “high school or less,” “associate or intermediate diploma,” “bachelor’s degree,” and “master’s or PhD”; occupation, which was grouped into “student,” “governmental worker,” “private worker,” and “unemployed”; marital status, “single,” “married,” or “other”; region of residence, included the 13 administrative regions of Saudi Arabia: “Riyadh,” “Makkah,” “Madinah,” “Qassim,” “Eastern Region,” “Asir Region,” “Tabuk,” “Hail,” “Northern Region,” “Jazan,” “Najran,” “Al-Baha,” and “Al-Jawf”; and nationality, “Saudi” and “Non-Saudi” within Saudi Arabia. Age and region categories were based on distributions from the GASTAT [30,39], and education levels followed the National Qualifications Framework 2023 [40].

### Pilot

In a preliminary phase of the study, we conducted a pilot to confirm the validity and relevance of this study prior to full implementation. We targeted a sample group representing 10% of the total study sample size, with 10 participants recruited from each of the four social media platforms, which are Instagram, X (Twitter), Snapchat, and TikTok, resulting in a total of 40 participants. Participants responded positively to the questionnaire, navigating it without difficulty. Based on feedback, we made two key adjustments to improve the survey. First, we added a “sometimes” response option to the question in the second domain, “Do you think social media is a good source for obtaining oral health information?” to provide a wider range of responses. Second, we simplified certain Arabic terms in the misconception domain to make the statements clear to participants. This pilot phase was conducted from June 17, 2024, to September 2, 2024. Participants involved in this pilot were not included in the final study sample.

### Statistical Analysis

Counts and percentages were reported to describe participants’ sociodemographic characteristics, their engagement with oral health information, and their self-reported oral health. We used the chi-square test to examine potential associations between the 3 domains and sex, identifying statistically significant relationships where applicable.

For the multivariable analysis, the independent variables included engagement with the oral health information domain (the manner of participants’ engagement with oral health–related information in social media measured via the six binary items, each item entered as a separate exposure in the model) and self-reported oral health (participants’ subjective evaluation of their oral health status measured via 3-point Likert scale). The dependent variables were defined as:

- Presence of oral health misconceptions (binary): had at least one misconception based on the 14 statements on oral health misconceptions.
- Number of oral health misconceptions (count): the total number of misconceptions identified per participant.

To assess these relationships, we used logistic regression models to present both crude (odds ratio) and adjusted odds ratio (AOR), with respective 95% CI, to examine engagement with oral health information and self-reported oral health (exposure variables) with the presence of any oral health misconceptions among social media users (outcome). Logistic regression was selected due to its appropriateness for modeling binary outcomes, allowing us to examine the likelihood of participants holding at least one misconception. Poisson regression is used to report the mean ratio and adjusted mean ratio (AMR) of oral health misconceptions (outcome) by the engagement with oral health information and self-reported oral health (exposure variables). Poisson regression was chosen because it is suitable for modeling count data, particularly when the outcome variable represents a frequency count of misconceptions per participant. We confirmed the appropriateness of the Poisson model by assessing equidispersion using the Pearson chi-square statistic, which indicated no overdispersion. Sex, education (as a measure of socioeconomic status), and nationality were selected and adjusted as potential confounders based on their theoretical relevance [41] and observed associations with both the exposure and the outcome in exploratory analyses.

A sensitivity analysis was performed for both regression models to assess the potential impact on the results if the response “don’t know” was reclassified as “agree” in the 14 statements addressing oral health misconceptions. We reclassified “don’t know” responses based on the assumption that these individuals might be more susceptible to misconceptions due to a lack of knowledge or confidence. This provided a more conservative, robust estimate of misconception prevalence, allowing us to examine the influence of uncertainty on our findings across different analyses. A 2-tailed α level of .05 was used to test relationships in both directions, and all statistical analyses were conducted using Stata/BE (version 17.0; StataCorp).

### Ethical Considerations

This study was conducted in compliance with the ethical standards set by the institutional review board of King Saud University. Research ethical approval was obtained, and the study was registered with the Institutional Review Board from the Committee of Human and Social Research Ethics, King Saud University (registration KSU-HE-23848). Participants were informed about the study objectives, procedures, and their rights before participating in the survey. Informed consent was obtained electronically via a mandatory “Agree” button at the start of the questionnaire. Only individuals who provided consent were able to proceed with the survey. The survey content was specifically designed to be low-risk without including sensitive or intrusive questions. Data were collected anonymously, with no personally identifiable information linked to the responses. The platform (SurveyMonkey) complied with strict data security and privacy protocols to ensure the protection of participants’ responses. IP addresses were monitored to identify duplicate entries within a 24-hour period and then omitted before analysis. Participation was voluntary, and no monetary or material compensation was provided to participants in this study. This study did not involve the use of images or media containing identifiable participants.

## Results

### Campaign Reach and Interaction Across Social Media Platforms

Using targeted social media campaigns across Instagram, X (Twitter), TikTok, and Snapchat, we observed notable differences in reach and interaction across platforms. TikTok achieved the highest reach (836,477) and link clicks (67,678) during its 30-day campaign, followed by Instagram, which reached (53,341) users and generated (1007) link clicks over 60 days. X (Twitter) and Snapchat had comparatively lower interactions. Across all platform advertisements, the overall view rate was 6.6%, and the participation rate was 0.7%.

### Sociodemographic Characteristics

Out of 640 individuals who initially responded to the questionnaire, 387 (76%) individuals completed it fully; the remaining (n=253) either provided incomplete responses or exited the survey after clicking “disagree” to participate in the questionnaire. These partial responses were excluded from the analysis to ensure data completeness.

The final sample size consisted of 248 (64%) female and 139 (35.9%) male participants (Table 1). The largest age group was 15-24 years (n=221, 57.1%), followed by 25-34 years (n=77, 19.9%). Most participants held a bachelor’s degree (n=184, 47.5%) or a high school diploma or less (n=138, 35.6%). Regarding occupation, more than half of the participants were students (n=199, 51.4%), while 97 (25%) participants were unemployed. In terms of marital status, 299 (77.2%) of participants were single, while 82 (21.1%) of participants were married. Saudi participants accounted for 72.3% (n=280) of the sample, and most participants resided in Riyadh (n=258, 66.6%). Significant sex-based differences were observed in occupation (*P*<.001) and marital status (*P*=.02), with male participants more likely to be employed and married compared to female participants, who were predominantly unemployed and single (Table 1).

**Table 1:** Sociodemographic characteristics and regional distribution among adults aged 15 years and older residing in Saudi Arabia (n=387), described by sex^a^.

Sociodemographic characteristics			Overall (n=387, 100%), n (%)		Male (n=139, 35.9%), n (%)		Female (n=248, 64%), n (%)		*P* value	
**Age (in years)**										.35
	15-24	221 (57.1)		78 (35.2)		143 (64.7)				
	25-34	77 (19.9)		22 (28.5)		55 (71.4)				
	35-44	45 (11.6)		19 (42.2)		26 (57.7)				
	45-54	36 (9.3)		16 (44.4)		20 (55.5)				
	55+	8 (2)		4 (50)		4 (50)				
**Education**										.23
	High school or less	138 (35.6)		49 (35.5)		89 (64.4)				
	Associate—intermediate diploma	39 (10)		15 (38.4)		24 (61.5)				
	Bachelor’s degree	184 (47.5)		61 (33.1)		123 (66.8)				
	Master’s degree—PhD	26 (6.7)		14 (53.8)		12 (46.1)				
**Occupation**										<.001
	Student	199 (51.4)		69 (34.6)		130 (65.3)				
	Governmental worker	43 (11.1)		24 (55.8)		19 (44.1)				
	Private worker	48 (12.4)		28 (58.3)		20 (41.6)				
	Unemployed	97 (25)		18 (18.5)		79 (81.4)				
**Marital status**										.02
	Single	299 (77.2)		98 (32.7)		201 (67.2)				
	Married	82 (21.1)		40 (48.7)		42 (51.2)				
	Other	6 (1.5)		1 (16.6)		5 (83.3)				
**Nationality**										.92
	Saudi	280 (72.3)		101 (36)		179 (63.9)				
	Non-Saudi	107 (27.6)		38 (35.5)		69 (64.4)				
**Region of residence**										.19
	Riyadh	258 (66.6)		84 (32.5)		174 (67.4)				
	Makkah	45 (11.6)		21 (46.6)		24 (53.3)				
	Madinah	13 (3.3)		7 (53.8)		6 (46.1)				
	Qassim	11 (2.8)		2 (18.1)		9 (81.8)				
	Eastern Region	45 (11.6)		21 (46.6)		24 (53.3)				
	Asir Region	5 (1.2)		1 (20)		4 (80)				
	Tabuk	1 (0.2)		0 (0)		1 (100)				
	Hail	2 (0.5)		0 (0)		2 (100)				
	Northern Region	1 (0.2)		0 (0)		1 (100)				
	Jazan	3 (0.7)		1 (33.3)		2 (66.6)				
	Najran	1 (0.2)		1 (100)		0 (0)				
	Al-Baha	1 (0.2)		1 (100)		0 (0)				
	Al-Jawf	1 (0.2)		0 (0)		1 (100)				

^a^Data were collected in a cross-sectional survey on oral health misconceptions conducted from September to November 2024.

### Engagement With Oral Health Information and Self-Reported Oral Health

Female participants were significantly more likely than male participants to use social media for oral health information (n=156, 68.7% compared to n=71, 31.2%; *P*=.02) and follow dental-specific accounts (n=126, 72% compared to n=49, 28%; *P*=.003; Table 2). For self-reported oral health, female participants were significantly more likely to rate their oral health as good (n=98, 73.1%) or average (n=125, 60.1%), while poor oral health was significantly slightly more common among male participants (n=20, 44.4%; *P*=.02).

**Table 2:** Engagement with oral health information and self-reported oral health described by sex among adults aged 15 years and older residing in Saudi Arabia (n=387)^a^.

Characteristics			Overall (n=387, 100%), n (%)		Male (n=139, 35.9%), n (%)		Female (n=248, 64%), n (%)		*P* value	
**Engagement with oral health information**										
	Use of social media to search for oral health information	227 (58.6)		71 (31.2)		156 (68.7)		.02		
	Ever received oral health information from nondental professionals on social media	235 (60.7)		86 (36.6)		149 (63.4)		.73		
	Following dental-specific accounts on social media	175 (45.2)		49 (28)		126 (72)		.003		
	Trust in oral health information from social media	171 (44.1)		54 (31.5)		117 (68.4)		.11		
	Social media profile content influence on trust in oral health information	289 (74.6)		105 (36.3)		184 (63.6)		.77		
	Perception of social media as a good source for oral health information	349 (90.1)		126 (36.1)		223 (63.9)		.82		
**Self-reported oral health**										.02
	Good	134 (34.6)		36 (26.8)		98 (73.1)				
	Average	208 (53.7)		83 (39.9)		125 (60.1)				
	Poor	45 (11.6)		20 (44.4)		25 (55.5)				

^a^Data were collected in a cross-sectional survey on oral health misconceptions conducted from September to November 2024.

### Patterns of Social Media Use

The patterns of social media use are summarized in Table 3. Of the total sample, the most frequently used platform on a daily basis was WhatsApp (n=344, 89.8%), followed by Instagram (n=304, 78.9%), Snapchat (n=239, 62%), and YouTube (n=196, 50.7%). The majority of our sample reported not using Line (n=374, 97.1%), Facebook (n=295, 76.4%), and LinkedIn (n=271, 70%). Telegram had a proportion of weekly users (n=134, 35.1%), while TikTok showed mixed use with 49.7% (n=192) reporting daily use and 31% (n=120) reporting no use.

**Table 3:** The patterns of social media use among adults aged 15 years and older residing in Saudi Arabia described by daily, weekly, monthly, and no use of commonly used platforms in Saudi Arabia^a^.

Social media platforms	Daily use^b^, n (%)	Weekly use^c^, n (%)	Monthly use^d^, n (%)	No use^e^, n (%)
X (Twitter)	180 (46.8)	98 (25.5)	37 (9.6)	69 (17.9)
Snapchat	239 (62)	49 (12.7)	11 (2.8)	86 (22.3)
WhatsApp	344 (89.8)	32 (8.3)	2 (0.5)	5 (1.3)
Instagram	304 (78.9)	52 (13.5)	9 (2.3)	20 (5.1)
TikTok	192 (49.7)	61 (15.8)	13 (3.3)	120 (31)
Facebook	32 (8.2)	29 (7.5)	30 (7.7)	295 (76.4)
Telegram	146 (38.3)	134 (35.1)	51 (13.3)	50 (13.1)
YouTube	196 (50.7)	155 (40.1)	21 (5.4)	14 (3.6)
Line	4 (1)	5 (1.3)	2 (0.5)	374 (97.1)
LinkedIn	26 (6.7)	47 (12.1)	42 (10.8)	271 (70.2)

^a^Data were collected in a cross-sectional survey on oral health misconceptions conducted from September to November 2024.

^b^Daily use: users who use the platform once a day or more than once a day.

^c^Weekly use: users who use the platform once a week, 2-3 times a week, or 4-6 times a week. ^d^Monthly use: users who use the platform once a month.

^e^No use: users who are not using the platform.

### Oral Health Misconceptions

Table 4 highlights the prevalence of oral health misconceptions among participants. Overall, 96.9% (n=375) of participants reported having at least one or more of the identified misconceptions. When stratified by sex, 93.5% (n=130) of male and 98.8% (n=245) of female participants were found to hold at least one or more misconceptions. Common misconceptions included “During pregnancy, the baby absorbs calcium from the mother’s teeth and bones” (n=337, 87%) and “Pregnant women should only undergo dental treatment after childbirth” (n=245, 63.3%). Additionally, 59.9% (n=232) of participants believed that “You should not eat anything when going for tooth extraction.” Sex-based differences were observed for the misconception that “Using a hard-bristled toothbrush makes teeth whiter,” with male participants more likely to agree (n=38, 57.5%) than female participants (n=28, 43.4%; Table 4).

**Table 4:** The proportion of responses to common oral health misconceptions among adults aged 15 years and older residing in Saudi Arabia, described by sex^a^.

Oral health misconceptions	Overall, n (%)	Male, n (%)	Female, n (%)
During pregnancy, the baby absorbs calcium from the mother’s teeth and bones	337 (87)	108 (32)	229 (67.9)
Pregnant women should only undergo dental treatment after childbirth	245 (63.3)	89 (36.3)	156 (63.6)
You should not eat anything when going for tooth extraction	232 (59.9)	90 (38.7)	142 (61.2)
Scaling weakens the structure of the teeth	224 (57.8)	80 (35.7)	144 (64.2)
Upper teeth extraction affects the brain	218 (56.3)	66 (30.2)	152 (69.7)
Brushing with salt helps whiten teeth	183 (47.2)	63 (34.4)	120 (65.5)
It is better not to brush your teeth when you have bleeding gums	157 (40.5)	69 (43.9)	88 (56)
Extracted teeth do not need to be replaced with artificial teeth	146 (37.7)	59 (40.4)	87 (59.5)
Taking antibiotics will relieve the tooth pain	137 (35.4)	50 (36.6)	87 (63.5)
If there is no tooth pain, there is no need to visit the dentist	130 (33.5)	51 (39.2)	79 (60.7)
Leaving a milk bottle in the baby’s mouth during sleep does not harm teeth	114 (29.4)	53 (46.4)	61 (53.5)
The appearance of wisdom teeth increases one’s wisdom	82 (21.1)	31 (37.8)	51 (62.2)
Using a hard-bristled toothbrush makes teeth whiter	66 (17)	38 (57.5)	28 (43.4)
There is no need to care for primary teeth, as they will be replaced by permanent teeth anyway	64 (16.5)	38 (59.3)	26 (40.6)
Having at least one or more of the above-mentioned misconceptions	375 (96.90)	130 (93.53)	245 (98.79)

^a^Data were collected in a cross-sectional survey on oral health misconceptions conducted from September to November 2024.

### Association Between Engagement With Oral Health Information and Self-Reported Oral Health With Oral Health Misconceptions

Logistic and Poisson regression analyses (Table 5) revealed significant associations between following dental-specific accounts on social media with lower odds of having any misconceptions (AOR 0.41, 95% CI 0.22-0.78) and a lower count of misconceptions (AMR 0.87, 95% CI 0.77-0.98). Trust in social media for oral health information was significantly associated with a higher count of misconceptions (AMR 1.16, 95% CI 1.02-1.31). Self-reported oral health showed that participants with poor oral health had a higher count of misconceptions (AMR 1.23, 95% CI 0.99-1.50), although the association was not statistically significant. After conducting the sensitivity analysis by combining “I don’t know” with “agree” as a single variable, there was no difference in most of the observations, except that participants who perceived social media as a good source of oral health information had a statistically significant higher count of misconceptions (AMR 1.18, 95% CI 1.02-1.37). This reclassification suggests that uncertainty about oral health information may reflect susceptibility to misconception.

**Table 5:** Logistic and Poisson regression models of the association between engagement with oral health information and self-reported oral health with the presence and count of oral health misconceptions among adults aged 15 years and older residing in Saudi Arabia^a^.

Characteristics				Any misconceptions					Number of misconceptions		
				Crude OR^b^ (95% CI)		Adjusted OR (95% CI)		Crude MR^c^ (95% CI)			Adjusted MR (95% CI)
**Engagement with oral health information**											
	**Use of social media to search for oral health information**										
		No	reference		reference		reference			reference	
		Yes	1.20 (0.67-2.16)		1.07 (0.59-1.97)		1.12 (0.99-1.28)			1.12 (0.99-1.28)	
	**Ever received oral health info from nondental professionals on social media**										
		No	reference		reference		reference			reference	
		Yes	1.33 (0.74-2.39)		1.33 (0.73-2.44)		1.01 (0.89-1.15)			1.00 (0.89-1.14)	
	**Following dental-specific accounts on social media**										
		No	reference		reference		reference			reference	
		Yes	0.49 (0.27-0.89)^d^		0.41 (0.22-0.78)^d^		0.85 (0.76-0.97)^d^			0.87 (0.77-0.98)^d^	
	**Trust in oral health information from social media**										
		No	reference		reference		reference			reference	
		Yes	1.81 (0.98-3.35)		1.68 (0.89-3.17)		1.16 (1.02-1.31)^d^			1.16 (1.02-1.31)^d^	
	**Social media profile content influence on trust in oral health information**										
		No	reference		reference		reference			reference	
		Yes	1.47 (0.79-2.77)		1.73 (0.89-3.35)		1.10 (0.95-1.28)			1.12 (0.97-1.30)	
	**Perception of social media as a good source for oral health information**										
		No	reference		reference		reference			reference	
		Yes	0.95 (0.35-2.55)		0.93 (0.34-2.58)		1.21 (0.97-1.51)			1.20, (0.96-1.50)	
**Self-reported oral health**											
	Good		reference		reference		reference			reference	
	Average		1.31 (0.71-2.42)		1.33 (0.70-2.53)		1.17 (1.01-1.34)			1.14 (0.99-1.31)	
	Poor		2.01 (0.65-6.19)		2.11 (0.66-6.76)		1.26 (1.03-1.54)			1.23 (0.99-1.50)	

^a^Data were collected in a cross-sectional survey on oral health misconceptions conducted from September to November 2024.

^b^OR: odds ratio.

^c^MR: mean ratio. Adjusted for sex, education, and nationality.

^d^Indicates statistical significance (*P*<.05).

## Discussion

### Main Findings

This study investigated the patterns of social media use and the prevalence of oral health misconceptions among Saudi adults, focusing on the relationship between engagement with oral health information on social media and self-reported oral health with oral health misconceptions. The study highlighted significant associations between following dental-specific accounts and lower oral health–related misconceptions, while higher trust in social media information was associated with a higher count of oral health–related misconceptions. Additionally, self-reported poor oral health was associated with higher counts of oral health–related misconceptions.

A significant portion of our sample consisted of students and women. The high proportion of students in our sample reflects that students are known to be highly active on social media and rely on these platforms for information seeking, including health-related information. This aligns with findings from a scoping review indicating that social media plays a dominant role in health education in Saudi Arabia and that younger individuals and women frequently seek medical and health-related advice through digital platforms [42].

The results of our targeted social media campaigns reveal distinct differences in audience reach and interaction across various platforms. TikTok, in particular, demonstrated a significant advantage in terms of reach. This aligns with previous studies that highlight TikTok’s rapid rise as a dominant platform for user interaction, especially among younger audiences [43,44]. Instagram, known for its visual-centric content, also proved effective in reaching a wide audience and generating meaningful interactions in this study. Instagram has been shown to be effective for health communication because of its ability to attract users through images, videos, and infographics, which enhance user interaction and retention [45]. In contrast, X (Twitter) and Snapchat showed lower interaction rates, which can be explained by their platform-specific characteristics. In contrast, both X (Twitter), with its focus on quick updates and real-time communication, and Snapchat, with its temporary content format appealing to younger users seeking informal and immediate interactions, limit their potential for sustained and broad interaction [46].

TikTok’s effectiveness in disseminating health information can be attributed to its short-form video format, which caters to modern users’ preference for quick, easily digestible content [47]. This format has not only captured the attention of younger audiences but also influenced other social media platforms to adopt similar features. The platform’s popularity among younger demographics further enhances its role in health communication, as it serves as a primary source of information for this age group. Moreover, TikTok’s participatory features, such as duets and challenges, encourage user participation and the viral spread of health-related content [48].

Our methodology assumed that using social media advertisement campaigns would effectively reach our target population, with advertisement reach aligning with the demographic distribution in Saudi Arabia, where men represent a larger proportion (61.2%) [49]. The term “ad recipient” refers specifically to individuals who were exposed to the advertisement (ie, saw the advertisement) without necessarily engaging with it by clicking. Men comprised the majority of advertisement recipients across all platforms: Snapchat (n=28,235, 56%), TikTok (n=625,665, 74.32%), Instagram (n=24,625, 57.2%), and X (Twitter; n=107,116, 61%), similar to the male demographic distribution in Saudi Arabia [49]. However, despite greater advertisement exposure among men, women represented the majority of questionnaire respondents. This difference in interaction may be explained by behavioral differences, as previous studies have found that women are more likely to interact with health-related advertisements and content on social media than men [50-52]. This trend may be driven by greater health awareness among women, a higher likelihood of managing family health matters, and cultural norms that encourage women to be more involved in health-related decision-making [53,54].

This study identified several common oral health–related misconceptions. The most prevalent misconceptions include the belief that pregnant women lose calcium during pregnancy, dental procedures should be postponed during pregnancy, eating before dental extractions is harmful, and scaling weakens tooth structure. These findings are consistent with earlier studies conducted in Saudi Arabia, which also documented widespread myths about oral health [26-28].

Many of these oral health misconceptions can negatively impact oral health by discouraging preventive behaviors and delaying necessary treatment. For example, myths about pregnancy and oral health, such as the belief that “Pregnancy causes calcium loss in teeth” or that “Dental treatment should be avoided during pregnancy,” may lead to untreated dental issues, increasing the risk of adverse pregnancy outcomes like preterm birth and low birth weight [55]. Similarly, misconceptions about primary teeth being unimportant contribute to early childhood caries, which affects children’s overall well-being and increases the likelihood of future dental problems [56]. Additionally, beliefs in ineffective home remedies, such as using salt for whitening or avoiding professional scaling, can lead to poor oral hygiene and a higher risk of periodontal disease [57,58]. Based on these observations, addressing these common oral health–related misconceptions through targeted public health campaigns and education is essential. Engaging the community to participate in the educational content, primarily through social media platforms, would be an active way of enhancing people’s awareness.

The high prevalence of misconceptions underscores the challenges in addressing oral health information in the digital age. While social media platforms provide an avenue for disseminating information, their effectiveness depends on the quality and credibility of the content shared.

This study observed associations between trust in social media for oral health information and higher misconceptions are consistent with broader concerns about the role of social media in propagating misconceptions in health communication [59]. The user-driven nature of social media echo chambers, combined with algorithm-driven content personalization, can reinforce existing beliefs, making users more susceptible to content that aligns with their biases and less likely to evaluate or seek out alternative perspectives critically. Social media algorithms on platforms such as TikTok, Instagram, and Facebook select content based on users’ past interactions, prioritizing posts that align with their preference history. As a result, users who have previously interacted with nonevidence-based health claims are more likely to be exposed to similar misconceptions [60,61]​. Conversely, the protective effect of following dental-specific accounts aligns with prior studies that emphasize the importance of verified content from credible sources in mitigating health misconceptions [62-65]. On social media, accounts maintained by professionals or organizations with expertise in a given field act as anchors of credibility, reducing the likelihood that users will engage with inaccurate information [59].

### Strengths and Limitations of the Study

Our research specifically investigates oral health–related misconceptions in Saudi Arabia, an area understudied despite social media’s growing influence in shaping health beliefs. This study’s multiplatform social media approach enabled a comprehensive analysis of social media’s role in oral health information. The use of targeted advertisements across multiple platforms, including Snapchat, TikTok, Instagram, and X (Twitter), allowed for the random selection of participants, enhancing the representativeness of the sample. We provided robust insights into the associations between engagement behaviors and self-reported oral health with oral health misconceptions by using advanced statistical modeling, including logistic and Poisson regression analyses. However, the cross-sectional design limits the ability to establish temporal relationships between the variables. Moreover, the reliance on self-reported data introduces potential response and recall biases, as participants may have overstated their engagement with oral health information or underreported misconceptions due to social desirability. Additionally, recall bias could have affected their ability to recall their pattern of social media use accurately. Furthermore, while we adjusted for key confounders, unmeasured variables may have influenced both the exposures and outcomes, potentially affecting the observed associations.

### Implication of the Study

Our findings align with previous research that highlights social media’s role as a valuable channel for disseminating health information and as a potential source of misconceptions [19,66]. Therefore, the highly used platforms like WhatsApp, Instagram, Snapchat, and YouTube, as observed in this study, highlight their potential as valuable tools for web-based dental public health campaigns in Saudi Arabia. These platforms are widely used daily, reflecting their integration into the community’s digital habits and their capacity to reach broad and diverse audiences. To address oral health–related misconceptions, health authorities, dental professionals, and regulatory bodies should consider the following:

- Developing social media–targeted campaigns that collaborate with credible, well-followed dental professional creators to disseminate accurate and engaging oral health information. This can include posting participatory videos, photos, and Q and A sessions to address common oral health–related misconceptions identified in our findings. Use social media features tailored to user interaction—such as TikTok’s targeting for reaching young adults, Instagram Reels for quick educational content, Snapchat Stories for oral health tips, and X (Twitter) threads to counter oral health–related misconceptions.
- Establishing Saudi health authorities verified social media pages that consistently share reliable oral health information, combat misconceptions, and engage with users. These dental-specific accounts should interact with trending dental topics to redirect audiences toward credible sources.
- Legalizing and monitoring both oral health–related advertisements and information in social media from Saudi regulatory bodies would help protect public health and ensure that individuals receive evidence-based information in social media. For example, the European Union’s Digital Services Act mandates that web-based platforms prevent the spread of misinformation, including health-related content, by enforcing fact-checking measures and transparency requirements for web-based advertisements [67].

While this study provides valuable insights, future research is needed to investigate how different content formats (eg, videos, photos, and participatory posts) and features (eg, likes, comments, and shares) influence the dissemination and retention of oral health information on social media. Experimental studies can assess which formats are most effective in improving knowledge and correcting oral health–related misconceptions, while longitudinal research can explore how repeated exposure to accurate content impacts behavioral change over time.

### Conclusions

The study underscores the role of social media in shaping oral health perceptions among adults in Saudi Arabia. Engagement with dental-specific accounts was associated with fewer misconceptions, while trust in oral health information in social media was associated with higher misconceptions. Platforms like WhatsApp, Instagram, TikTok, and Snapchat are widely used in Saudi Arabia and offer the potential for targeted dental public health campaigns. Strategic collaborations with credible dental professional creators to disseminate accurate and engaging oral health information. Creating Instagram reels for quick educational content or using TikTok’s targeting features could enhance information retention and user interaction. Although this study focuses on Saudi Arabia, its findings have broader relevance, particularly in regions with high social media penetration and similar digital health-seeking behaviors, such as the Gulf countries. From a policy perspective, legalizing and monitoring oral health–related advertisements and information in social media should be implemented by regulatory bodies to protect public health.

Future research should build on these findings to investigate the effectiveness of varying social media platforms’ content formats and features in improving oral health knowledge and correcting misconceptions through experimental studies. Longitudinal research should also explore the long-term impact of repeated exposure to accurate information on behavioral change. These insights provide a basis for stakeholders to formulate evidence-based, innovative strategies to improve public health in the context of the digital age.

## Appendix

Multimedia Appendix 1 Questionnaire used in the study to assess social media usage patterns, engagement with oral health information, self-reported oral health, oral health misconceptions, and sociodemographic characteristics among adults in Saudi Arabia.

## Abbreviations

AMR: adjusted mean ratio
AOR: adjusted odds ratio
GASTST: General Authority of Statistics

## Data Availability

The datasets generated or analyzed during this study are not publicly available due to ethical restrictions and commitments that mandate strict confidentiality of participant information and prohibit the use of data for purposes beyond the approved research study outlined by the institutional review board at King Saud University, but are available from the corresponding author on reasonable request.
