# Supplementary material for: Social Media Use and Oral Health–Related Misconceptions in Saudi Arabia: Cross-Sectional Study
Source: JMIR Form Res. 2025 Feb 10;9:e70071. doi: 10.2196/70071 (PMC11851047; doi:10.2196/70071)
Supplement: Multimedia Appendix 1 [file formative_v9i1e70071_app1.docx]

**Dear Participant,**

This questionnaire is part of a doctoral student’s research in the field of dental public health - from the College of Dentistry at King Saud University. The aim of the study is to assess the use of social media platforms and their relationship to oral health-related information in Saudi Arabia and to explore your opinion about some oral health-related information. Kindly answer the following questions to the best of your ability. The questionnaire is composed of five parts and needs approximately 5 minutes to be completed.

Your information, confidentiality, and anonymity will be protected. Only the researcher will have access to your information and responses.

If you have any queries or comments, please do not hesitate to contact the investigator of the study at

[rahafbinhamdan@gmail.com](mailto:rahafbinhamdan@gmail.com)

**Do you agree to participate in this questionnaire?**

- **I agree**
- **I do not agree**

**Part 1: The patterns of social media use:**

1. Please select your favorite social media platform and indicate your frequency of use:

| **Preferred Platform** | **Frequency of Use** | | | | | | |
| --- | --- | --- | --- | --- | --- | --- | --- |
| Twitter | More than once a day | Once a day | 4-6 times a week | 2-3 times a week | Once a week | Once a month | I do not use this platform |
| Snapchat | More than once a day | Once a day | 4-6 times a week | 2-3 times a week | Once a week | Once a month | I do not use this platform |
| WhatsApp | More than once a day | Once a day | 4-6 times a week | 2-3 times a week | Once a week | Once a month | I do not use this platform |
| Instagram | More than once a day | Once a day | 4-6 times a week | 2-3 times a week | Once a week | Once a month | I do not use this platform |
| Facebook | More than once a day | Once a day | 4-6 times a week | 2-3 times a week | Once a week | Once a month | I do not use this platform |
| TikTok | More than once a day | Once a day | 4-6 times a week | 2-3 times a week | Once a week | Once a month | I do not use this platform |
| Telegram | More than once a day | Once a day | 4-6 times a week | 2-3 times a week | Once a week | Once a month | I do not use this platform |
| YouTube | More than once a day | Once a day | 4-6 times a week | 2-3 times a week | Once a week | Once a month | I do not use this platform |
| Line | More than once a day | Once a day | 4-6 times a week | 2-3 times a week | Once a week | Once a month | I do not use this platform |
| LinkedIn | More than once a day | Once a day | 4-6 times a week | 2-3 times a week | Once a week | Once a month | I do not use this platform |

**Part 2: Engagement with oral health information:**

1. Do you use social media to search for oral health information?

- Yes
- No

1. Have you ever received oral health-related information from non-dental professionals in social media?

- Yes
- No

1. Do you follow any dental-specific accounts on social media?

- Yes
- No

1. Do you trust any oral health-related information you receive from social media?

- Yes
- No

1. Does the profile content (e.g., personal photo, bio, design....etc.) of dental-specific accounts in social media affect your decision to trust the provided oral health information?

- Yes
- No

1. Do you think that social media is a good source for obtaining oral health-related information?

- Yes
- Sometimes
- No

**Part 3: Self-reported oral health:**

1. In general, how would you rate your oral and dental health?

- Good
- Average
- Poor

**Part 4: Oral health-related information:**

**The following are statements related to oral health. We would like to know your opinion on whether you agree, disagree, or are don’t know. Please answer the following questions based on your personal experience. We encourage you to respond honestly, as all your answers are confidential and highly valuable.**

1. If there's no tooth pain, there's no need to visit the dentist:

- Agree
- Disagree
- Don’t know

1. . Scaling (cleaning the teeth in the dental clinic by using an electronic device to remove calculus) will weaken the tooth structure:

- Agree
- Disagree
- Don’t know

1. The appearance of wisdom teeth increases one's wisdom:

- Agree
- Disagree
- Don’t know

1. Cleaning your teeth using a hard bristled toothbrush makes your teeth whiter:

- Agree
- Disagree
- Don’t know

1. Brushing your teeth with salt helps to whiten your teeth:

- Agree
- Disagree
- Don’t know

1. You should not eat anything when you are going for tooth extraction:

- Agree
- Disagree
- Don’t know

1. Extracted teeth do not need to be replaced with artificial teeth:

- Agree
- Disagree
- Don’t know

1. Extraction of the upper teeth will affect the brain:

- Agree
- Disagree
- Don’t know

1. Pregnant women should only undergo dental treatment after childbirth:

- Agree
- Disagree
- Don’t know

1. It is better not to brush your teeth when you have bleeding gums:

- Agree
- Disagree
- Don’t know

1. During pregnancy, the baby absorbs calcium from the mother's teeth and bones:

- Agree
- Disagree
- Don’t know

1. Leaving a milk bottle in the baby's mouth during sleep does not harm teeth:

- Agree
- Disagree
- Don’t know

1. If you have pain in a particular tooth, taking antibiotics will relieve the tooth pain:

- Agree
- Disagree
- Don’t know

1. There is no need to care for primary teeth, as they will be replaced by permanent teeth anyway:

- Agree
- Disagree
- Don’t know

**Part 5: Sociodemographic information:**

1. Age:

- 15-19
- 20-24
- 25-29
- 30-34
- 35-39
- 40-44
- 45-49
- 50-54
- 55-59
- 60-64
- 65 or above

1. Sex:

- Male
- Female

1. Education level:

- Primary education
- Intermediate education
- Secondary education
- Associate Diploma - Intermediate Diploma
- Bachelor’s degree
- Master’s degree - Ph.D. degree

1. Occupation:

- Student
- Work in the governmental sector
- Work in the private sector
- Do not work

1. Marital status:

- Single
- Married
- Others:……………….

1. Nationality:

- Saudi
- Non-Saudi:…………….

1. Region of residence:

- Riyadh
- Makkah
- Madinah
- Qassim
- Eastern Region
- Asir Region
- Tabuk
- Hail
- Northern Region
- Jazan
- Najran
- Al-Baha
- Al-Jawf

**Thank You**
